# Biochemical and immunohistochemical examination of the effects of ephedrine in rat ovary tissue

**DOI:** 10.1590/acb381523

**Published:** 2023-05-01

**Authors:** Veysel Toprak, Senem Alkan Akalın, Ece Öcal, Yunus Çavuş, Engin Deveci

**Affiliations:** 1 Diyarbakir Memorial Hospital – Division of Gynecology and Obstetrics – Diyarbakir, Turkey; 2 Private Medical Practice – Division of Gynecology and Obstetrics – Diyarbakir, Turkey.; 3 Private Medical Practice – Division of Perinatology – Diyarbakir, Turkey.; 4 Dicle University – Faculty of Medicine – Department of Histology and Embryology – Diyarbakır, Turkey.

**Keywords:** Interleukin-6, Caspase 3, Antioxidants, Apoptosis

## Abstract

**Purpose:**

It was aimed to investigate the biochemical and immunohistochemical effects of ephedrine (EPH) in bilateral ovariectomized rats.

**Methods:**

Twenty-four Sprague Dawley female rats were divided into three groups: control group: The abdomen was opened and closed without any treatment; ischemia-reperfusion (IR) group: 2 h of ischemia followed by 2 h of reperfusion were allowed to cause IR injury; IR+EPH group: oral EPH solution (5 mg/kg) was administered for 28 days.

**Results:**

Biochemical parameters were statistically significant in group comparisons. Increased interleukin-6 (IL-6) expression, degenerative preantral and antral follicle cells and inflammatory cells around blood vessels were seen in IR group. Negative IL-6 expression was observed in seminal epithelial cells, preantral and antral follicle cells in IR+EPH group. While caspase-3 activity increased in granulosa cells and stromal cells in IR group, caspase-3 expression was negative in preantral and antral follicle cells in the germinal epithelium and cortex in IR+EPH group.

**Conclusions:**

The effect of apoptosis, which occurs with the signaling that starts in the cell nucleus, caused the cessation of the stimulating effect at the nuclear level after EPH administration, and a decrease in the antioxidative effect in IR damage and inflammation in the apoptotic process.

## Introduction

The removal of ovaries is called ovariectomy (OVX), and this procedure causes decreased estrogen levels and bone loss, namely osteoporosis^1^. Osteoporosis is verified by lower bone mineral density and lower trabecular number and thickness as well as higher trabecular separation observed in studies with experimental animals. These changes are observed at 14, 30, and 60 days post-OVX in proximal tibia, lumbar vertebrae, and femur, respectively^2^. An important point in the pathogenesis of osteoporosis is decreased estrogen release^3^. The widely used is the 12-week-old female Sprague Dawley rat models in which osteoporosis and bone development were studied with OVX^4^.

Ephedrine (C_10_H_15_NO, EPH) is an active α- and β-adrenergic agonist that can be used orally and has a long-lasting effect. EPH creates its effect by directly and indirectly affecting adrenergic receptors. The indirect effect is caused by the release of noradrenaline from adrenergic nerve endings. The effects of EPH are various in organs. The first action is by bronchodilation with β-2 adrenergic stimulation; it increases vital capacity and relieves respiration. EPH stimulates the central nervous system and contracts the bladder smooth muscles. EPH increases the contractility and conduction power of myocardium with β-1 adrenergic stimulation and causes an increase in pulse rate and heart rate volume. EPH causes elevation in blood pressure and nasal decongestion. After oral administration of 25 mg, bronchodilation occurs after 0.25-1 h and lasts for 3-5 h. EPH is rapidly absorbed from the gastrointestinal tract after oral administration and undergoes minimal metabolism in the liver and excreted via the kidneys^5^^-^ 7. EPH is usually used in nonsevere asthma, to control chronic asthma rather than to treat acute seizures. EPH is also used in the symptomatic treatment of reversible bronchospasms associated with chronic bronchitis, emphysema and bronchospastic respiratory disorders^8^. EPH has been shown to increase energy expenditure and cause the loss of body fat in rats and mice^9^. Interleukin-6 (IL-6) is a cytokine secreted from cells such as macrophages, T cells, stromal and osteoblastic cells, and it can show significant antioxidative effects^10^. It can stimulate or inhibit cell growth, regulate cell differentiation, and increase metastasis by promoting cell adhesion and/or tumor angiogenesis^11^. Caspase-3 is the key that plays a role in the progression of the pro-apoptotic process towards apoptosis as a result of inflammation and leads to cell-to-cell by degrading DNA repair proteins^12^.

This study aimed to investigate the biochemical and immunohistochemical changes resulting from the administration of EPH for 28 days in rats that underwent bilateral OVX.

## Methods

### Animals

All experimental procedures were approved by the Dicle University Animal Experimentation Local Ethics Committee. Experimental animals were obtained from Dicle University Health Sciences Research and Application Center.

### Surgical procedure

Rats were given general anesthesia with 90 mg/kg intramuscular ketamine hydrochloride and 8 mg/kg xylazine before starting the surgical procedure. Female Sprague Dawley rats of 12-weeks-old (n = 24) were used. Rats were housed in clean cages with free access to water and food, in room temperature with a 12/12 h of dark and light cycle. In order to provide analgesia, 25 mg/kg metamizole sodium (Devaljin) was administered twice a day for the first two days to all operated groups.

### Experimental groups

Control group: No treatment was applied to animals. Only the abdomen was opened with a surgical protocol and the abdominal folds closed without any further intervention.

Ischemia-reperfusion (IR) group: The abdominal area opened with a 2-cm midline incision. Ischemia was created for 2 h with a disposable Bulldog clamp on ovarian tissues. Then, the ovaries were placed back to their normal positions, in their anatomical location, and the blood flow will be reperfused for 2 h.

IR+EPH group: The same protocol applied in the 2nd group was applied in this group but, received EPH (99%, CAS no: 134-71-4, Sigma) for 28 days. At the end of the experimental procedures, rats were sacrificed by excessive xylazine (Rompun; Bayer, Istanbul, Turkey) injection and ovary tissues were removed immediately.

### Biochemical analysis

Malondialdehyde (MDA) levels and glutathione (GSH) activities were determined in the ovaries of each rat, and the average values of each group were calculated. Each ovary sample was prepared as a 10% homogenate (according to weight) in 0.9% saline using a homogenizer on ice. Then, the homogenate was centrifuged at 2,000 rpm for 10 min, and the supernatant was collected. MDA levels were determined using the double heating method of Draper et al.^13^. MDA values were expressed as nanomoles per gram (nmol/g) of wet tissue. GSH activity was measured by the method of Paglia et al.^14^. An enzymatic reaction was initiated by the addition of hydrogen peroxide (H_2_O_2_) to a tube that contained reduced nicotinamide adenine dinucleotide phosphate, reduced glutathione, sodium azide, and glutathione reductase. The change in absorbance at 340 nm was monitored by spectrophotometry. Superoxide dismutase (SOD) and catalase (CAT) determination was done according to Oslvik et al.^15^. Data were expressed as U/g.

### Tissue processing

Dissected ovarian tissues were fixed in formaldehyde solution and dehydrated through increased alcohol series and incubated in paraffin wax. Samples were embedded in paraffin blocks. Five μm ovarian sections were cut for the immunohistochemical analysis. Sections were deparaffinized in xylene solution, passed through decreased alcohol series and washed in distilled water^16^.

### Immunohistochemical analysis

Formaldehyde-fixed tissue was embedded in paraffin wax for further immunohistochemical examination. Sections were deparaffinized in 70, 80, 90 and 96% alcohols. The antigen retrieval process was performed twice in citrate buffer solution (pH 6.0), first for 10 min, and second for 7 min, boiled in a microwave oven at 700 W. They were allowed to cool to room temperature for 15 min and washed twice in distilled water for 5 min. Endogenous peroxidase activity was blocked in 0.1% H_2_O_2_ (catalogue #TA-015-HP, Thermo Fisher Scientific, USA) for 20 min. Blocking solution (TA-125-UB, Thermo Fisher Scientific, USA) was applied for 10 min prior to the application of primary antibodies, which were left on overnight caspase-3 antibody (1:100 dilution) and IL-6 (1:100 dilution) .The sections were washed three times for 5 min in phosphate buffered saline (PBS) and then were incubated with biotinylated secondary antibody for 25 min. After washing with PBS, streptavidin peroxidase was applied to the sections for 25 min. The sections were washed three times for 5 min in PBS. Diaminobenzidine was applied to the sections for up to 15 min as a chromogen. The control slides were prepared using the same procedure, without primary antibodies. Counterstaining was done using Harris’s hematoxylin for 45 s, dehydrated through ascending alcohol and cleared in xylene (Product Number: HHS32 Sigma, hematoxylin solution, Harris modified, Sigma-Aldrich, 3050 Spruce Street, Saint Louis, MO, 63103, USA). The slides were mounted with Entellan (lot: 107961, Sigma-Aldrich, St. Louis, MO, USA) and examined under a light microscope (Olympus, Germany)^17^^,^ 18.

### Statistical analysis

The data were recorded as arithmetic mean ± standard deviation with mean rank value. Statistical analysis was done using the IBM SPSS 25.0 software (IBM, Armonk, New York, USA). Kruskal–Wallis test was used for multiple comparisons. Within-group comparisons, Mann–Whitney U and were used. P < 0.05 was used as the significance level.

## Results

Statistical analysis of biochemical and histochemical parameters was shown in Table 1. MDA, IL-6 expression, and caspase-3 expression were statistically higher in IR group than control group. SOD and CAT activity in IR group were lower than control group and this decrease was statistically significant. In IR+EPH group, MDA, IL-6 expression, and caspase-3 expressions were statistically decreased, and SOD and CAT activity content were statistically increased in IR+EPH group compared to IR group. In this study, control, IR and IR+EPH groups were compared. Compared to the control group, the MDA value was found to be higher in the IR group, but the MDA value in the IR+EPH group was close to the control group. It was thought that the antioxidative effect of EPH administration in lipid peroxidation is important. In addition, while SOD and CAT values were low in the IR group, reaching values close to the control in IR+EPH group strengthened the antioxidative effect.

**Table 1:** Biochemical and Immunohistochemical analysis of Control, IR and IR+EPH groups.

Parameters	Groups	n	Median (Min-Max)	Kruskal-Wallis		Mann-Whitney U Test (p<0.05)
				Mean Rank	H test P value	
MDA	(1) Control	8	5.96 (5.34–6.98)	7.88	15.485 P < 0.001	(2)
	(2) IR	8	9.79 (9.11–12.44)	20.50		(1) (3)
	(3) IR+EPH	8	6.12 (5.86–6.56)	9.13		(2)
SOD	(1) Control	8	3.58 (2.99–3.97)	16.25	15.380 P < 0.001	(2)
	(2) IR	8	1.23 (1.02–1.77)	4.50		(1) (3)
	(3) IR+EPH	8	3.56 (2.23–4.22)	16.75		(2)
CAT	(1) Control	8	0.05 (0.04–0.06)	15.50	15.375 P < 0.001	(2)
	(2) IR	8	0.02 (0.01–0.03)	4.50		(1) (3)
	(3) IR+EPH	8	0.05 (0.04–0.06)	17.50		(2)
GSH	(1) Control	8	381.87 (348.63–398.21)	18.25	16.340 P < 0.001	(2)
	(2) IR	8	288.45 (242.24–315.85)	4.50		(1) (3)
	(3) IR+EPH	8	369.66 ( 359.82–392.22)	14.75		(2)
IL-6 expression	(1) Control	8	1.00 (0.00–2.00)	6.69	15.227 P < 0.001	(2)
	(2) IR	8	3.00 (2.00–4.00	19.88		(1) (3)
	(3) IR+EPH	8	1.50 (0.00–3.00	10.94		(2)
Caspase-3 expression	(1) Control	8	1.00 (0.00–2.00	7.50	15.777 P < 0.001	(2)
	(2) IR	8	3.00 (2.00–4.00	20.19		(1) (3)
	(3) IR+EPH	8	1.00 (1.00–2.00	9.81		(2)

Multiple statistical comparisons between the IR group and the Control and IR+EPH groups were significant according to the Mann–Whitney U Test. P < 0.05 was used as the significance level. (1: Control; 2: IR; 3: IR+EPH).

## Immunohistochemical findings

### IL-6 expression

**Figure 1:**
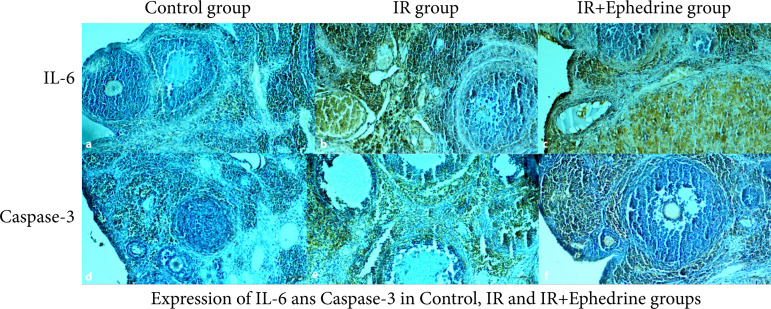
IL-6 and Caspase-3 immunostaining in Control, IR and IR+EPH groups. **(a)** IL-6 expression in Control group; **(b)** IL-6 expression in IR group; **(c)** IL-6 expression in IR+EPH group; **(d)** Caspase-3 expression in Control group; **(e)** Caspase-3 expression in IR group; **(f)** Caspase-3 expression IR+EPH group (Bar: 100 μm, magnification: 10×).

In Control group, IL-6 expression was weak and negative in the granulosa cells in the preantral and antral follicles. In the theca follicular sheath and in the connective tissue cells in the stromal region, IL-6 expression was positive in a few inflammatory cells around some small blood vessels (Fig. 1a). In IR group, IL-6 expression was positive in degenerative preantral, antral follicle cells, cells in the corpus luteum, and connective tissue cells in the stromal region. In addition, increased IL-6 expression was observed in aggregated and solitary dispersed inflammatory cells around blood vessels (Fig. 1b). In IR+EPH group, IL-6 expression was positive in some degenerated follicle cells in the germinal epithelial cells, while IL-6 expression was negative in the preantral and antral follicle cells. And, IL-6 expression was negative in the endothelial cells in the blood vessels and in the solitary connective tissue cells around the vessel. But, IL-6 expression was found to be moderate in cells in the corpus luteum (Fig. 1c).

### Caspase-3 expression

In Control group, caspase-3 immunoactivity was found to be negative in preantral and antral follicle cells, and caspase-3 expression was moderate in some stromal cells and fibroblast cells (Fig. 1d).

In IR group, caspase-3 activity was increased in degenerative, granulosa, and stromal cells in preantral and antral follicles. Caspase-3 reaction was positive in expanding blood vessel endothelial cells and cells around degenerative basement membrane (Fig. 1e).

IR+EPH group, negative caspase-3 expression was seen in preantral and antral follicle cells in the germinal epithelium and cortex region, and caspase-3 expression in many areas in stromal cells showed a positive reaction (Fig. 1f).

## Discussion

A sympathomimetic agent EPH is known to be used in treatment of hypotension and as a bronchodilator for symptomatic treatment of asthma and as a nasal decongestant. In bronchospasm and for the treatment of mild symptoms of asthma, EPH is used in doses of 12.5–25 mg and for hypotension during anesthesia in doses of 25–50 mg in human subjects (FDA). Considering the related adverse effects and the risk potential, rats were received EPH 5.0 mg/kg/day^19^. In a study on juvenile rats that were administered doses of 2, 10, or 60 mg/kg EPH sulfate (i.v.) daily from postnatal day 35 to 56, mortality has accelerated at dose of 60 mg/kg. The no-adverse-effect level was 10 mg/kg (almost 1.9 times a maximum daily dose of 50 mg in a 60 kg person based on body surface area).

Arbo et al.^19^ administered EPH 5.0 mg/kg/day and p-synephrine 50.0 mg/kg/day to estrogen-treated females’ rats. Here, macroscopical alterations were evaluated in liver, kidneys, adrenals, and uterus. All analyzed substances showed an antiestrogenic potential, but only EPH at 0.5 mg/kg/day presented a significative antiestrogenic effect (P < 0.01). As a result of applied ischemia or a toxic substance, cellular defense against oxidative damage, antioxidant enzymes such as SOD, CAT and GSH are formed^20^. Increasing ROS production and antioxidant consumption change the oxidative-antioxidative balance in favor of oxidative stress^21^. In the ovarian IR study performed by Ermiş et al.^10^, it was shown that MDA value was high, SOD, GSH, CA value was low after IR, and these values came to levels close to control with the use of antioxidant effective losartan. In this study, the Control, IR and IR+EPH groups were compared, and the MDA value was found to be higher in the IR group compared to the control group, but the MDA value was close to the control group in IR+EPH group (Table 1).

It is thought that the antioxidative effect of EPH application is important in lipid peroxidation. In addition, while SOD, GSH, CAT values were low in the IR group, reaching values close to the control in IR+EPH group shows the antioxidative effect. Lipid peroxidation results in the development of an inflammatory response due to changes in ionic channel lipids and increased permeability to calcium and other ions^22^.

Cytokines such as IL-1-β and IL-6 from blood vessel endothelial cells and phagocytic mononuclear cells are rapidly synthesized and released into circulation during ischemia and IR, along with increased oxidative stress and inflammation during IR blood flow deceleration and oxygen restriction. Nuclear structure induced by inactivation of NF-kβ signaling pathways after IR induced the expression of inflammatory factors including TNF-α, IL-1β and IL-6 and increased apoptosis. In the IR group, it was observed that IL-6 expression increased with increasing inflammation and apoptotic changes began to occur with the emergence of proapoptotic cells.

Caspases are a family of genes maintaining homeostasis through regulating cell death and inflammation. They participate in ordered processes such as apoptosis and inflammation. Caspases are classified according to their roles in apoptosis; caspase-3acts as an executioner caspase^23^. Sapmaz et al.^24^ showed that IR injury did not reduce the number of ovarian germ cells, but increased granulosa cell death. They investigated that it prevents the development of follicles by applying caspase-3 with the tunnel method. In another study, a significant increase in caspase-3 activation was observed in granular cells in mature antral follicles and inflammatory cells in the stromal region in the ischemia group, while they showed negative expression of caspase-3 expression in antral follicle cells and granular cells around the antral follicle in the application of antioxidants such as rosmarinic acid^25^. After IR, caspase-3 activity increased in preantral and antral follicle, granulosa, and stromal cells, and apoptotic effect increased in blood vessel endothelial cells and connective tissue cells around the basement membrane.

Limitations were that western blot could be performed to support quantitatively immunohistochemical staining.

## Conclusion

The initiation of degenerative changes in preantral and antral follicle cells in the ovary, as well as in stromal cells and blood vessel endothelial cells due to oxygen deficiency after IR indicates an increase in the inflammation signal and acceleration of the proapoptotic process. The effect of apoptosis, which occurs with the signaling that starts in the cell nucleus, caused the stimulatory effect to pause at the nuclear level after EPH administration, and the antioxidative effect in IR injury, and the inflammation apoptotic process decreased.

## Data Availability

All data sets were generated or analyzed in the current study.
